# A longitudinal survey of African animal trypanosomiasis in domestic cattle on the Jos Plateau, Nigeria: prevalence, distribution and risk factors

**DOI:** 10.1186/1756-3305-6-239

**Published:** 2013-08-19

**Authors:** Ayodele O Majekodunmi, Akinyemi Fajinmi, Charles Dongkum, Kim Picozzi, Michael V Thrusfield, Susan C Welburn

**Affiliations:** 1 Division of Pathway Medicine and Centre for Infectious Diseases, School of Biomedical Sciences, College of Medicine and Veterinary Medicine, The University of Edinburgh, Chancellor’s Building, 49 Little France Crescent, Edinburgh EH16 4SB, UK; 2 Nigerian Institute for Trypanosomiasis Research, P.M.B. 1303, Vom, Plateau State, Nigeria; 3 Veterinary Clinical Sciences, Royal (Dick) School of Veterinary Studies, College of Medicine and Veterinary Medicine, Easter Bush Veterinary Centre, Roslin, Midlothian EH25 9RG, UK

**Keywords:** Animal African Trypanosomiasis, *Trypanosoma congolense*, *Trypanosoma vivax*, *Trypanosoma brucei brucei*, Prevalence, Risk factors, Seasonal dynamics, PCR, Jos Plateau, Nigeria

## Abstract

**Background:**

Trypanosomiasis is a widespread disease of livestock in Nigeria and a major constraint to the rural economy. The Jos Plateau, Nigeria was free from tsetse flies and the trypanosomes they transmit due to its high altitude and the absence of animal trypanosomiasis attracted large numbers of cattle-keeping pastoralists to inhabit the plateau. The Jos Plateau now plays a significant role in the national cattle industry, accommodating approximately 7% of the national herd and supporting 300,000 pastoralists and over one million cattle. However, during the past two decades tsetse flies have invaded the Jos Plateau and animal trypanosomiasis has become a significant problem for livestock keepers.

**Methods:**

In 2008 a longitudinal two-stage cluster survey on the Jos Plateau. Cattle were sampled in the dry, early wet and late wet seasons. Parasite identification was undertaken using species-specific polymerase chain reactions to determine the prevalence and distribution bovine trypanosomiasis. Logistic regression was performed to determine risk factors for disease.

**Results:**

The prevalence of bovine trypanosomiasis (*Trypanosoma brucei brucei, Trypanosoma congolense* savannah*, Trypanosoma vivax*) across the Jos Plateau was found to be high at 46.8% (39.0 – 54.5%) and significant, seasonal variation was observed between the dry season and the end of the wet season. *T. b. brucei* was observed at a prevalence of 3.2% (1% – 5.5%); *T. congolense* at 27.7% (21.8% - 33.6%) and *T. vivax* at 26.7% (18.2% - 35.3%). High individual variation was observed in trypanosomiasis prevalence between individual villages on the Plateau, ranging from 8.8% to 95.6%. Altitude was found to be a significant risk factor for trypanosomiasis whilst migration also influenced risk for animal trypanosomiasis.

**Conclusions:**

Trypanosomiasis is now endemic on the Jos Plateau showing high prevalence in cattle and is influenced by seasonality, altitude and migration practices. Attempts to successfully control animal trypanosomiasis on the Plateau will need to take into account the large variability in trypanosomiasis infection rates between villages, the influence of land use, and husbandry and management practices of the pastoralists, all of which affect the epidemiology of the disease.

## Background

Agricultural development is essential for growth across sub-Saharan Africa, employing 65% of the labour force and accounting for 32% of gross domestic product [1]. Diseases of livestock reduce agricultural output by up to 30% in developing countries (twice the impact as in developed countries) [2]. The majority of the disease burden faced is from infection with endemic diseases, in particular African Animal Trypanosomiasis (AAT), tick borne diseases and helminthiases, all of which decrease production and increase morbidity and mortality. The presence of AAT is estimated to reduce cattle density by 37 – 70%, reduce off take by 50%, reduce the calving rate and increase calf mortality by 20% [3].

The Jos Plateau has previously been considered free of AAT, the altitude being assumed to be too high to permit tsetse colonisation and rendering the Plateau free of the tsetse vector and the trypanosomes they transmit [4,5]. Consequently, the plateau has been used in various predictive models to set the current limits for areas habitable for tsetse and used to predict future limits. The risk maps generated have been used to identify possible disease clusters, to define and monitor outbreaks, to target control measures and resources and to follow changes in disease patterns over space and time across Sub Saharan Africa [6-9]. On closer examination it is clear that both tsetse flies and AAT have been present on the Jos Plateau at least since the 1980s from reports from single village surveys and from surveys undertaken in Local Government Areas and that these assumptions are no longer valid [10]. To date there have been no robust studies that have attempted to define overall prevalence of AAT and trypanosome species distributions across the Jos Plateau. Using a combination of molecular diagnostics and socio-economic surveys of cattle keepers, this study presents the first empirical assessment of the current status of AAT across the Jos Plateau.

## Methods

Quantitative and qualitative methods were applied in a three-phase study to determine seasonal variations in the prevalence of AAT across the Jos Plateau between March and October 2008.

### Study site

The study area comprised the Jos Plateau, situated in North Central Nigeria between latitude 9.2422°N - 10.1153°N and longitude 8.6957°E - 9.5210°E. The Jos Plateau comprises an area of 8000 km^2^ with an average altitude of 1280 m, lying at the centre of four major river basins: Lake Chad to the North; Benue to the South; Gongola to the east and Kaduna to the west. The annual rainfall is between 1000 -1500 mm and many small streams originate on the plateau and drain into each of the four river basins. The rainy season is between May and October, lasting between 160–220 days and the dry season runs from November to April.

### Study design

**Figure 1:**
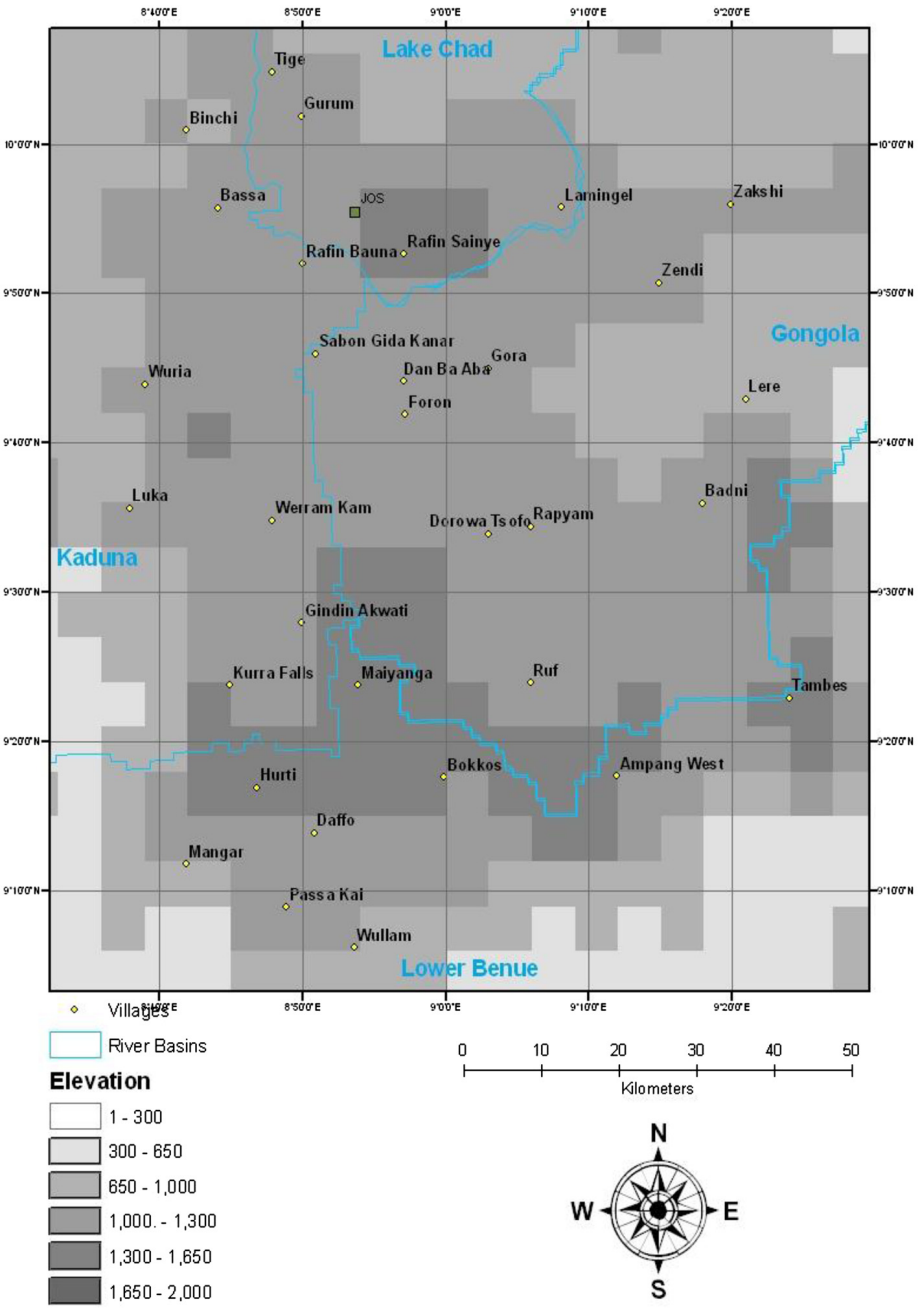
The Jos Plateau showing selected villages.

A longitudinal two-stage stratified cluster sampling design was applied in which individual cattle represented the ultimate sampling unit and individual villages were clusters. In the first stage, villages were stratified by their river basin and selected using probability proportional to size. For the second stage, a fixed number of cattle were selected in each village that was to be included in the study. An assumed mean trypanosomiasis prevalence of 24% [11-14] and rate of homogeneity of 0.115 [15] were applied and entered into CSurvey (©UCLA, 2007) to determine the minimum number of clusters required and to test the final sample size. A minimum number of thirty individual clusters (villages), with eighty cattle sampled per cluster were required to estimate AAT disease prevalence with a confidence level of 95%. Thirty villages were selected by applying a 15 km × 20 km grid across the study area, with one village per square identified as indicated in Figure 1.

Following an assessment of the available scientific literature concerning seasonal variations in tsetse and trypanosomiasis migration patterns on the Jos Plateau in particular and more generally in northern Nigeria [16] a three-point longitudinal survey was designed to examine seasonal variations in AAT whereby sampling for AAT was undertaken during the dry season (March), early wet season (June) and the late wet season (October).

Eighty cattle were randomly selected for sampling from individual herds within each village. Blood was collected from each animal by venipuncture and drawn blood applied directly onto Whatman FTA™ cards. FTA Cards were air-dried at room temperature for at least one hour and stored in sealed envelopes with desiccant prior to processing for PCR analysis. FTA cards were used to seed PCR reactions to determine AAT prevalence [17].

### PCR methodology

Five individual 3mm discs were excised from each card for each individual animal sampled using a Harris Micropunch© (Whatman, UK). To avoid cross contamination between samples, five discs were punched from blank filter cards after each sample. The five 3 mm discs of blank filter paper were included as negative controls for the DNA extraction process. The FTA discs were washed twice for 15 minutes using 1ml of Whatman FTA purification reagent to remove haemoglobin, discarding used reagent after each wash. FTA cards were then washed twice for 15-minutes in TE buffer (10 mM Tris, 0.1 mM EDTA, pH 8.0) to remove the FTA purification reagent and again the used buffer was discarded after each wash. FTA discs were dried for 30 minutes in an oven at 37°C. Chelex suspension (100 μl of 5%) was added to the dry discs and discs were incubated at 90°C for 30 minutes to elute DNA from the FTA discs. Eluted DNA was used to seed subsequent PCR reactions being found to be more sensitive than using a dried FTA disc as recommended by the manufacturers [18-20].

Three individual species-specific PCR reactions were undertaken to detect the trypanosome species that are most commonly found in cattle in Nigeria: *T. b. brucei*, *T. congolense* savannah and *T. vivax*. PCR amplifications were carried out in 25 μl reaction mixtures containing 10 × RedTaq reaction buffer (670 mM Tris–HCl pH 8.8, 166 mM (NH4)2SO4, 4.5% Triton X-100, 2 mg/ml gelatin) (Sigma Aldrich), 2 mM MgCl2, 200 μM of each of the four deoxynucleoside triphosphates (dNTPs), primers at 1 μM and 1U of RedTaq DNA polymerase (Sigma Aldrich) and 5 μl sample DNA.

DNA was amplified using a Dyad Peltier thermal cycler© (MJ Research Inc. USA). The following primer sets were used to identify individual trypanosome species: 

*T. b. brucei*[21]

TBR1: 5′CGAATGAATAAACAATGCGCAGT3′

TBR2: 5′AGAACCATTTATTAGCTTTGTTGC3′

*T. congolense* savannah [22]

TCS 1: 5′CGAGAACGGGCACTTTGCGA3′;

TCS 2: 5′GGACAAACAAATCCCGCACA3′

*T. vivax*[23]

ILO 1264: 5′CAGCTCGCCGAAGGCCACTTGGCTGGG–3′;

ILO 1265: 5′– TCGCTACCACAGTCGCAATCGCAATCGTCGTCTGAAGG– 3′.

PCR was carried out using an initial step of 94°C for 3 minutes, followed by 35 cycles of 94°C for 1 minute, 55°C for 1 minute, 72°C for 30 seconds, and a final extension of 72°C for 5 minutes. 15 μl of the PCR product was run on a1.5% agarose gel stained with GelRed© (Biotium, USA).

### Qualitative methods

A structured questionnaire, incorporating participatory rural appraisal (PRA) techniques was completed with all livestock owners who participated in the AAT survey to investigate the effect of cattle movement on prevalence of AAT disease. The relationship between presence of trypanosomiasis at the individual animal level and several predictor variables including altitude, migration and alien cattle were explored by logistic regression [24] using Egret for Windows© (Cytel Software, 1999). A mixed effect, multivariate, additive risk model was constructed with fixed effect variables as potential risk factors for which information was available from questionnaires (see Table 1). A village was included as a random effect within the model to account for any clustering by village. Exact 95% binomial confidence intervals [25] were calculated using CIA© BMJ software. Exact binomial confidence intervals were also calculated for differences between proportions and these differences were considered significant when their confidence intervals did not contain zero [26,27]. The Moran’s I test for spatial correlation [28] was used to identify spatial clustering of trypanosomiasis at 5% level of significance using ARCGIS© ESRI software.

**Table 1:** Logistic regression variables

**Variable**	**Type of variable**	**Class of variable**
**Trypanosomiasis**	Outcome variable	Binomial
		positive = 1, negative = 0
**Altitude**	Predictor variable	Categorical, 12 categories
		range 800 m – 1350 m
		50 m increase per category
**Dry season migration**	Predictor variable	Binomial
		yes = 1, no = 0
**Wet season migration**	Predictor variable	Binomial
		yes = 1, no = 0
Alien migratory cattle*	Predictor variable	Binomial
		yes = 1, no = 0

*cattle domiciled elsewhere migrating along the cattle routes on the Jos Plateau.

### Ethical approval statement

The study was carried out with the full approval of cattle keepers, the Plateau State Ministry of Agriculture and the Nigerian Institute for Trypanosomiasis Research (NITR), a Federal Government body. The University of Edinburgh is a charitable body, registered in Scotland, with registration number SC005336.

## Results

### AAT prevalence

A total of 7143 individual cattle were sampled in 30 villages across the three sampling periods and in total 46.8% of them were positive for AAT (27.7% were infected with *T. congolense,* 26.7% with *T. vivax* and3.2%with *T. b. brucei*)as shown in Table 2.

**Table 2:** Prevalence for AAT on the Jos Plateau (95% confidence intervals in brackets)

	**Dry Season**	**Early wet season**	**Late wet season**	**Annual**
***T. b. brucei***	3.0%	5.3%	1.4%	3.2%
	(2.4% - 3.9%)	(4.4% - 6.2%)	(0.9% – 1.9%)	(2.8% – 3.6%)
***T. congolense***	30.7%	25.1%	25.6%	27.7%
	(22.8% - 38.6%)	(15.5% - 34.7%)	(17.0% - 34.1%)	(21.8% - 33.6%)
***T. vivax***	24.8%	22.1%	29.9%	26.7%
	(13.7% – 36.0%)	(10.8% - 33.5%)	(17.8% - 42.1%)	(18.2% - 35.3%)
**Trypanosomiasis**	44.9%	43.8%	47.9%	46.8%
	(33.1% - 56.7%)	(31.7% - 56.0%)	(37.5% - 58.4%)	(39.0 – 54.5%)

During the dry season (March) 2330 cattle were sampled across the 30 villages and 44.9% of them were found to be positive for AAT comprising 30.7% infected with *T. congolense*, 24.8% infected with *T. vivax* and 3.0% harbouring *T. b. brucei* infections. During the early wet season (June), 2449 cattle were sampled and 43.8% of them were positive for AAT comprising 25.1% with *T. congolense*, 22.1% infected with *T. vivax* and 5. 3 % infected with *T. b. brucei*. In the late wet season (October) a total of 2367 cattle were sampled and 47.9% of them were positive for trypanosomiasis comprising 25.6% infected with *T. congolense,* 29.9% infected with *T. vivax* and 1.4% infected with *T. b. brucei*. Significant seasonal variation was observed in trypanosomiasis prevalence between the dry season in March and the late wet season in October (see Table 2) showing a proportional difference of 3.0% (0.16 – 5.9%). *T. brucei* infection is highest in the early wet season; infection with *T. congolense* in highest in the dry season and *T. vivax* infection highest in the late wet season.

**Figure 2:**
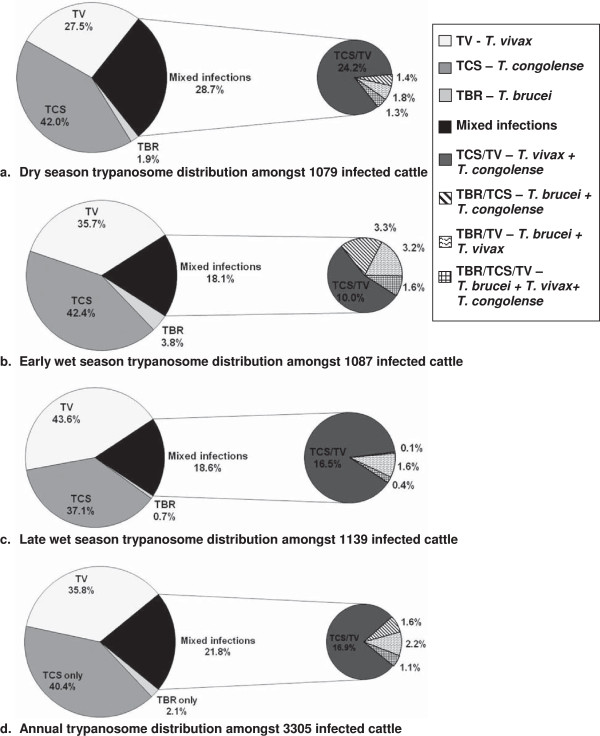
The distribution of trypanosome species amongst infected animals.

The proportion of animals harbouring infections of multiple species was observed to change with season (see Figure 2). The majority of mixed infections observed in cattle were for *T. congolense* and *T. vivax*. Figure 2 shows proportions of single and mixed infections amongst AAT positive cattle. The greatest proportion of mixed infections in cattle were observed during the dry season at 24.2%, falling to 18.1% in the early wet season and 18.6% during the late wet season. The greatest proportion of animals infected with *T. b. brucei* and either or both of *T. congolense* and *T. vivax* was found in the early wet season.

**Figure 3:**
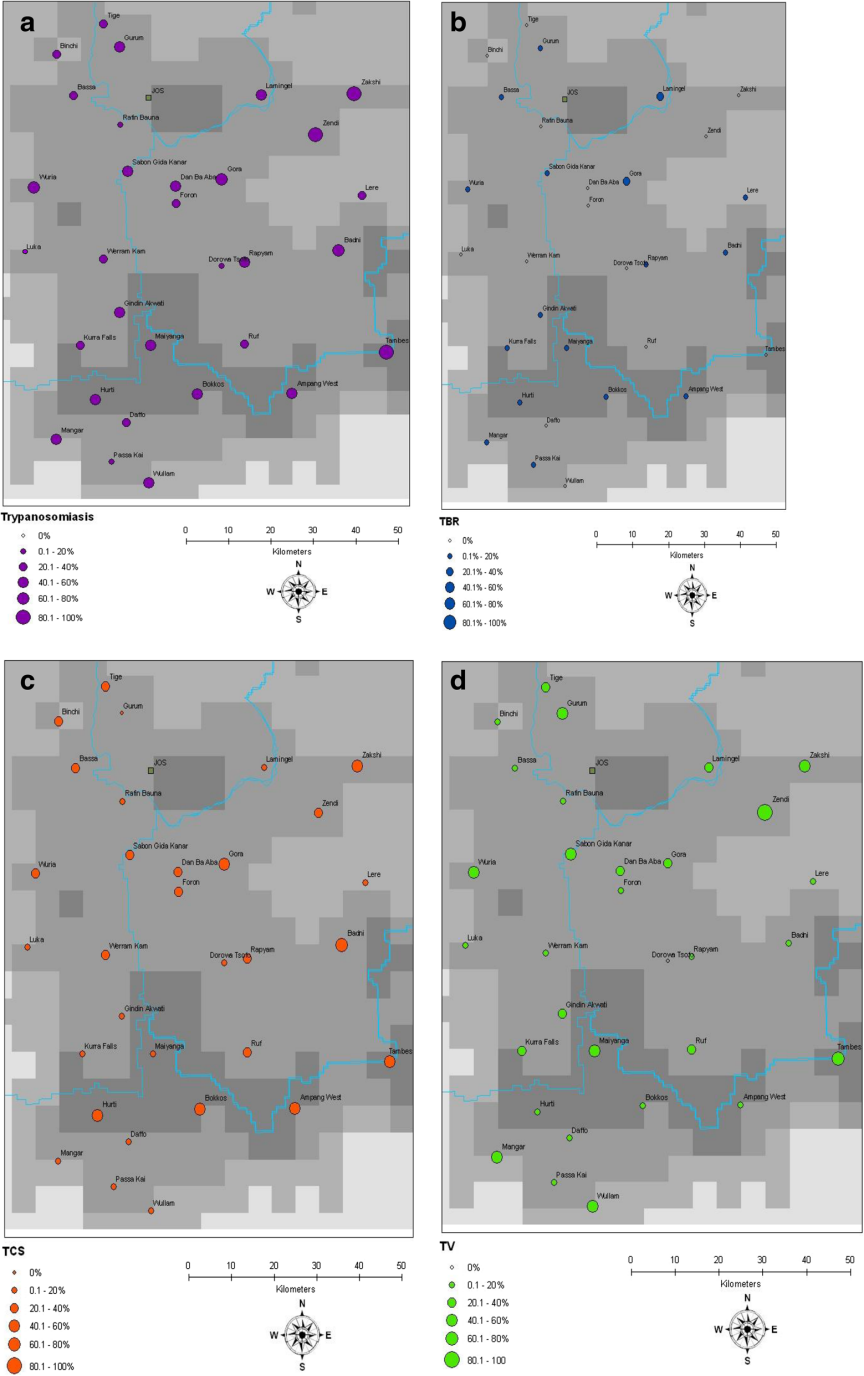
**Prevalence and distribution of AAT across sampled villages (annual): (a) AAT (b) *T. *b*. brucei *(c) *T.congolense *savannah (d) *T. vivax.***

There was no evidence of geographical clustering of bovine trypanosomiasis that could be detected on the Jos Plateau (Moran’s I = 0.01, p = 0.902). At village level the total AAT prevalence ranged from 8.8% (5.8% - 13.0%) to 95.6% (91.3% - 97.9%) as observed in Figure 3a. For individual trypanosome species, village level prevalence ranged from 2.6% - 62.1% for *T. congolense* (Figure 3b), from 0% – 95.6% for *T. vivax* (Figure 3c) and from 0% - 22.2% for *T. b. brucei* (Figure 3d).

### AAT risk factors

Cattle migration, both the dry and wet seasons showed weak positive association to trypanosomiasis prevalence, with odds ratios of 1.22 (1.01 – 1.37) and 1.23 (1.09 – 1.37) respectively (see Table 3). Altitude, showed a strong negative association for AAT with an odds ratio of 0.91 for every 50 m increase in altitude as compared to cattle kept at 800 m. The risk of an animal acquiring an AAT infection is 0.91 times less at 850 m and 0.35 times less at 1350 m. The presence of migratory cattle from outwith the study area showed a weak negative correlation with trypanosomiasis with an odds ratio of 0.70 (0.62 – 0.77). All odds ratios are close to one and odds ratios less than three are not considered sufficient evidence of a causal relationship [29].

**Table 3:** Logistic regression output for risk factors for trypanosomiasis

**Variable**	**P value**	**Odds ratio (95% CI)**
**Altitude**	< 0.001	0.91 (0.90 – 0.93)
**Dry season migration**	< 0.001	1.22 (1.01 – 1.37)
**Wet season migration**	0.0253	1.23 (1.03 – 1.47)
**Alien migratory cattle**	< 0.001	0.70 (0.62 – 0.77)

## Discussion

### Trypanosomiasis prevalence

The evidence presented here shows that AAT is now endemic across the Jos Plateau. The overall prevalence of trypanosomiasis across the Jos Plateau was considerable at 46.8% (39.0 – 54.5%), and these levels of infection would be expected to be detrimental to cattle health and productivity. Prevalences of between 37.6% and 40% have previously been recorded from individual village surveys of the Jos Plateau, using less sensitive detection methods but were conducted in response to outbreaks of disease [12,30,31]. Classically, infection with the trypanosome species that are pathogenic in local breeds of cattle, *T. congolense* and *T. vivax*, results in retarded growth and anaemia and an animal’s nutritional status is a key factor determining the outcome of any infection [32-34]. The proportion of infected animals showing clinical signs of infection will depend on their condition and nutritional status; in this study, the cattle were generally of poor nutritional status due to a prolonged dry season, poor access to pasture in the wet season and lack of feed supplementation. Local Fulani breeds are clearly able to maintain high levels of AAT infection even when under considerable nutritional stress.

A high level of variability was observed in village level prevalences of AAT over the 30 villages selected for screening across the Plateau, ranging from 8.8% (5.8% - 13.0%) to 95.6% (91.3% - 97.9%). Such high variation in village level prevalence and the observed absence of geographical clustering of disease across this wide area suggest that environmental factors on the Plateau have relatively little effect impact on the epidemiology of the disease but rather village specific factors such as microhabitats, land use patterns and husbandry practices play a major role in determining AAT infection in cattle.

The variation in trypanosomiasis prevalence observed between the dry season in March and late wet season in October (proportional difference 3.0% {0.16 – 5.9%}), is consistent with expected seasonal variations in tsetse populations and distribution in Nigeria [16,35]. The dry season in March is associated with low humidity and high temperatures, which reduce fecundity and increase mortality in the two tsetse species found on the Plateau, *Glossina tachinoides* and *Glossina palpalis palpalis*, limit tsetse dispersal within the environment and reduce transmission of AAT; by contrast, climatic conditions in the wet season support increased tsetse populations, greater tsetse dispersal and increased transmission of AAT [36,37].

The low prevalence of *T. brucei* parasites (3.2%) is consistent with previous surveys carried out on the Jos Plateau [12,38], across Nigeria in general [39,40] and elsewhere in Africa in cattle [41-43], and in sheep and goats [44]. The low prevalence in cattle may relate to the reported resistance of indigenous West African cattle to *T. brucei* infections [38]. Both tsetse species found at low densities on the plateau: *G. tachinoides* and *G. p. palpalis*[10], are able to transmit *T. brucei* but transmit this species at a lower frequency [45].

The prevalence of *T. congolense* in cattle across the Jos Plateau was found to be 27.7% (21.8% - 33.6). *T. congolense* contributes the major proportion of the AAT infectious burden on the Plateau, representing 60% of all AAT infections identified. Classically, *T. vivax* has been considered responsible for most of the cases of cattle trypanosomiasis in West [4] and also on the Jos Plateau [46,47]. Most previous studies have used parasitological techniques that have low specificity for differentiating trypanosome strains to determine prevalence [48]; although *T. vivax* is slightly larger and more motile than *T. congolense*, it is not always straightforward to differentiate ‘slow *T. vivax*’ in a blood sample from an active *T. congolense* parasite. Takeet and others [49] similarly show that where both microscopy and PCR are applied, microscopy erroneously identifies *T. vivax* as the most prevalent species and fails to identify *T. congolense*. Since *T. congolense* is more pathogenic than *T. vivax* for cattle [34], the high prevalence observed here has serious implications for animal health and productivity. The prevalence of *T. vivax* was also high at 26.7% (18.2% - 35.3%) but consistent with previous results in this area [13,30,31,39].

### Risk factors for trypanosomiasis

Altitude was found to be a risk factor for AAT. Each 50 m increase in altitude reduces risk of trypanosomiasis by 0.91 (0.90 – 0.93). Cattle kept at the highest altitudes on the Plateau have 0.35 times less risk of trypanosomiasis than those kept at the lowest altitudes. The altitude of the Jos Plateau ranges from between 1,200 – 1,777 m, believed to be close to the limits for tsetse survival at 1,800 m [50]. The higher altitude zones of the Plateau tend to be more highly populated and intensively farmed, resulting in fragmented tsetse habitats that may reduce AAT transmission.

Results indicated that dry season migration increased the risk of trypanosomiasis infection by 1.22 (1.01 – 1.37) while wet season migration increased risk by 1.23 (1.09 – 1.37). Fulani routinely undertake migration from the Jos Plateau, to areas with more abundant food and water, leaving young calves and nursing cows behind. Cattle may experience increased tsetse challenge when *en route* on migration and at particular destinations where there is increased vector/host contact; migration may increase cattle susceptibility, reducing animal condition due to stresses resulting from trekking and migration, including exposure to other vector borne diseases. When trucks replaced trekking as the mode of transport for cattle to be traded at Jos market, the point prevalence of AAT in cattle was reduced from 5.3% to 0%. This was even more pronounced in cattle traded at the southern market of Ilorin in the humid zone that showed a decrease in AAT from 65% to 8.5% [51].Extensive livestock management is a known risk factor for AAT with reported prevalence in extensively managed cattle (14.4%) being almost double those reported in intensively managed animals in Nigeria(7.45%) [38].

The presence of cattle in a village that are migrating along the cattle routes across the Jos Plateau from other parts of Nigeria i.e. alien migratory cattle in a village, reduce the risk of AAT by 0.70 (0.62 – 0.77). Alien migratory cattle may act as a buffer between tsetse and local cattle, being more likely to be bitten by tsetse flies since they are camped outside the village in open spaces rather than kept closer in with the local cattle. The transient presence of alien migratory cattle in a village may reduce the number of tsetse bites on local cattle and reduces their risk of AAT infection. Widespread movements of alien cattle to a new region that settle in the new area have been shown to have serious consequences for immigration of both human and animal parasites in previously disease free regions of Uganda [52].

It is clear that the Jos Plateau is neither free of tsetse not AAT. It is likely that the Sahelian droughts of the 1970s and 1980s that caused significant cattle migrations and human movements were responsible for the introduction of tsetse flies and trypanosomiasis to the Jos Plateau. The 1968–1974 droughts led to the loss of 300,000 animals and reduced agricultural yields by 60% in Northern Nigeria. The 1982–1986 droughts resulted in the loss of 5 million tonnes of grain, at least 120,000 animals from conflicts, severe constraints on biological productivity, and forced migrations [53,54]. The coping strategy of pastoralists across the Republic of Niger and Northern Nigeria during these droughts was to migrate to Southern Nigeria to seek pasture where they remained for several years before returning North [55-58]. The northward mass movement of returning cattle carried tsetse populations with it [50] and this migration is likely to have introduced flies and AAT on to the Jos Plateau that lies along a major West African cattle highway that stretches from Chad to Mali. Once on the Plateau, stable populations of tsetse established, transmitting AAT. The earliest reports of AAT on the Jos Plateau date from 1982 [59]. Infected migratory cattle were implicated in AAT outbreaks on the Plateau in 1996 [30,31]. The associations between migration and trypanosomiasis on the Plateau described here illustrate a similar effect albeit weaker since the range of contemporary migrations is less and the differences in prevalence between parasite species are more homogeneous.

## Conclusion

Bovine trypanosomiasis is now endemic on the Jos Plateau where cattle are predominantly infected with the cattle pathogens *Trypanosoma congolense* and *T. vivax*. This longitudinal survey of bovine trypanosomiasis showed a high overall prevalence of trypanosomiasis 46.8% (39.0 – 54.5%) with high variation between villages (8.8% - 95.6%). Seasonal variation was evident showing increases in AAT prevalence from 44.9% (33.1% - 56.7%) in the dry season to 47.8% (37.5% - 58.4%) by the end of the wet season. Altitude was a significant risk factor for trypanosomiasis and migration also influences AAT risk. Given the large variability in AAT infection rates between villages, attempts to successfully control animal trypanosomiasis in this area will need to take into account the influence on AAT epidemiology of local animal husbandry, land use and management practices across the Plateau. An understanding of the social factors affecting seasonal variation in bovine trypanosomiasis on the Jos Plateau may serve to facilitate adoption of sustainable control practices [Majekodunmi AO, Fajinmi A, Dongkum C, Picozzi K, Thrusfield MV, Welburn SC: Social factors affecting seasonal variation in bovine trypanosomiasis on the Jos Plateau, Nigeria submitted].

## Competing interests

The authors declare they have no competing interests and the sponsors had no role in the study design, data collection and analysis, decision to publish, or preparation of the manuscript.

## Authors’ contributions

Conceived and designed the experiment: AM, KP, CD, SCW, MT. Performed the experiment: AM, AF and CD carried out sample collection in the field. Analyzed the data: AM, SCW, MT. Contributed reagents/materials/analysis tools: AM, KP, AF, CD, MT, SCW. Wrote the paper: AM, SCW. All authors read and approved the final manuscript.
